# Global genotype flow in *Cercospora beticola* populations confirmed through genotyping-by-sequencing

**DOI:** 10.1371/journal.pone.0186488

**Published:** 2017-10-24

**Authors:** Niloofar Vaghefi, Julie R. Kikkert, Melvin D. Bolton, Linda E. Hanson, Gary A. Secor, Scot C. Nelson, Sarah J. Pethybridge

**Affiliations:** 1 School of Integrative Plant Science, Plant Pathology & Plant-Microbe Biology Section, Cornell University, Geneva, New York, United States of America; 2 Cornell Cooperative Extension, Canandaigua, New York, United States of America; 3 United States Department of Agriculture–Agricultural Research Service (USDA-ARS), Red River Valley Agricultural Research Center, Fargo, North Dakota, United States of America; 4 Department of Plant Pathology, North Dakota State University, Fargo, North Dakota, United States of America; 5 USDA-ARS, Sugar Beet and Bean Research Unit, Michigan State University, Michigan, United States of America; 6 College of Tropical Agriculture and Human Resources, Department of Tropical Plant and Soil Sciences, University of Hawaii at Manoa, Honolulu, Hawaii, United States of America; National Cheng Kung University, TAIWAN

## Abstract

Genotyping-by-sequencing (GBS) was conducted on 333 *Cercospora* isolates collected from *Beta vulgaris* (sugar beet, table beet and swiss chard) in the USA and Europe. *Cercospora beticola* was confirmed as the species predominantly isolated from leaves with Cercospora leaf spot (CLS) symptoms. However, *C*. cf. *flagellaris* also was detected at a frequency of 3% in two table beet fields in New York. Resolution of the spatial structure and identification of clonal lineages in *C*. *beticola* populations using genome-wide single nucleotide polymorphisms (SNPs) obtained from GBS was compared to genotyping using microsatellites. Varying distance thresholds (bitwise distance = 0, 1.854599 × 10^−4^, and 1.298 × 10^−3^) were used for delineation of clonal lineages in *C*. *beticola* populations. Results supported previous reports of long distance dispersal of *C*. *beticola* through genotype flow. The GBS-SNP data set provided higher resolution in discriminating clonal lineages; however, genotype identification was impacted by filtering parameters and the distance threshold at which the multi-locus genotypes (MLGs) were contracted to multi-locus lineages. The type of marker or different filtering strategies did not impact estimates of population differentiation and structure. Results emphasize the importance of robust filtering strategies and designation of distance thresholds for delineating clonal lineages in population genomics analyses that depend on individual assignment and identification of clonal lineages. Detection of recurrent clonal lineages shared between the USA and Europe, even in the relaxed-filtered SNP data set and with a conservative distance threshold for contraction of MLGs, provided strong evidence for global genotype flow in *C*. *beticola* populations. The implications of intercontinental migration in *C*. *beticola* populations for CLS management are discussed.

## Introduction

Understanding the genetic structure and evolutionary trajectory of pathogen populations in agroecosystems is fundamental to sustainable disease management [1]. Population genetics studies provide insights into pathogen biology, epidemiology, and co-evolutionary interactions with plants, which, in turn, help define management units and dynamic management strategies in response to ever-changing pathogen populations [1,2]. Recently, microsatellites (also known as simple sequence repeats) have been the markers of choice for population genetics studies, and have improved our knowledge of the biology and epidemiology of plant pathogens [3–8]. Some of the characteristics that make microsatellites popular (high mutation rate and variability) also complicate data analysis due to homoplasy, null alleles and complex mutation patterns that may violate assumptions of mutation-migration-drift equilibrium [9,10]. Moreover, ascertainment bias, caused by selection of the most polymorphic microsatellite loci after screening a limited number of individuals, may introduce a systematic bias in the estimates of population variation and structure [11,12]. Some studies also have proposed that microsatellites do not accurately reflect genome-wide patterns of diversity [13,14].

Alternatively, single nucleotide polymorphisms (SNPs) offer genome-wide coverage; and confer stable inheritance and analytical simplicity [15]. However, the biallelic nature and lower information content of SNPs demand a higher number of loci to be analyzed, which has traditionally hindered the use of SNPs in population genetics studies of non-model organisms [16–18]. Moreover, ascertainment bias persists as a substantial complication in methods of SNP discovery that use a non-representative sample of individuals for discovery of loci that are subsequently used for genotyping a broader set of individuals [16].

The advent of reduced-representation sequencing (RRS) methods [19] that facilitate genome-wide discovery of a large number of SNPs at lower costs has enabled the transition from population genetics to population genomics [20,21]. RRS approaches such as restriction site associated DNA sequencing (RAD-seq) [22] and genotyping-by-sequencing (GBS) [23] use restriction enzymes to reduce genome complexity before sequencing. These methods enable sequencing of a targeted genome fraction for a large number of individuals thereby reducing ascertainment bias through simultaneous marker discovery and genotyping [12,14]. Population genomics approaches can improve population genetics studies through generating multitudes of polymorphic markers that better reflect the genome-wide genetic diversity of populations [13,24]; and enhance resolution to identify fine-scale genetic variation [25–27] or detect rare recombination events [28].

An essential step in population genomics analyses using RRS genotyping techniques is filtering of poor quality reads and loci with low read depth, low minor allele frequency and a high proportion of missing data [20]. However, best filtering practices are poorly defined for population genomics studies [20,21]. Another potential area of confusion when using RRS genotyping techniques, especially for population genomics analyses of clonal populations, is the identification of genotypes and clones [20,29]. When using only a handful of microsatellite loci, a clone is defined as a unique multi-locus genotype (MLG) [30,31]. However, sequencing and SNP calling errors and missing data in RRS genotyping techniques result in identification of genetically identical individuals as different MLGs [25,29,32]. Moreover, in populations where clonal reproduction predominates, accumulation of somatic mutations over generations results in genetically non-identical individuals within clones, which may be more readily differentiated through high throughput genotyping methods with improved resolution. Thus, assignment of clones based only on MLG will result in highly inflated estimates of clonal diversity [12,20]. Presence of genotyping errors or somatic mutations at high frequencies may be detected through a peak at very low distance magnitudes in the frequency distribution of genetic distances. This may be used to define a distance threshold below which distinct MLGs are assembled into multi-locus lineages (MLL) [32]. Use of analytical tools that depict clonal boundaries based on defined genetic distance thresholds is, therefore, indispensable for identification of MLLs in genome-wide SNP data sets [20,29,32].

*Cercospora beticola* Sacc. is a haploid fungus and the cause of Cercospora leaf spot (CLS) on *Beta vulgaris* L. (sugar beet, table beet and swiss chard) worldwide. Previous population genetic studies using microsatellite markers uncovered cryptic recombination and high genetic diversity [33–38] in *C*. *beticola* populations, yet also revealed the predominance of multiple clones in New York and Hawaii [37–38]. Discovery of low population differentiation and distribution of recurrent genotypes across table beet fields separated by kilometers was interpreted as evidence for long distance dispersal of *C*. *beticola* in its asexual form, *i*.*e*., genotype flow [38]. Since asexual spores of *C*. *beticola* are reported to be dispersed by water or wind over short distances [39,40], long distance dispersal of clones may be mediated by other mechanisms such as agricultural machinery [41,42], seedborne inocula [34,43,44], or insects [39,43]. The relative roles of these mechanisms in initiation of CLS epidemics are not yet fully understood.

Detection of genotype flow in microsatellite population genetics studies of *C*. *beticola* may also have been a function of the low power of the 12, microsatellite loci to discriminate non-identical genotypes. In addition, due to the high mutation rate of microsatellite loci, identical multi-locus genotypes may arise through convergent evolution (homoplasy), and not be identical by descent [12]. The use of molecular markers with lower mutation rates, *e*.*g*., SNPs, and more refined genotyping methods such as GBS that provide thousands of markers and greater resolution of clonal structure may improve our understanding of genotype flow in *C*. *beticola* populations.

The primary objective of this study was to investigate the occurrence of global genotype flow in *C*. *beticola* populations using microsatellites [29,33] and GBS [23]. We used the R package *poppr* [29,45] for defining clonal boundaries in a GBS-SNP data set of *C*. *beticola* populations, and assessed the impact of various filtering strategies and distance thresholds on the estimation of clonal diversity and genotype flow. A complementary objective was to compare measures of clonal diversity, differentiation and structure of *C*. *beticola* populations obtained from both genotyping approaches.

## Materials and methods

### Fungal isolates and species identification

In total, 333 *Cercospora* isolates sampled from *B*. *vulgaris* (sugar beet, table beet and swiss chard) in the USA and Europe were included in this study (Table 1). The populations from Hawaii (Diamond Head community garden) and New York (Farms 1 and 2, Fields 3 and 5) were described in a previous study [38]. In brief, the Hawaiian population consisted of 67 isolates collected from swiss chard and table beet growing in sympatry in a community garden in Honolulu, Hawaii. The New York populations were collected from two mixed-cropping farms (Farms 1 and 2) and two monoculture table beet fields (Fields 3 and 5 planted to cultivars ‘Ruby Queen’ and ‘Red Ace’, respectively). The mixed-cropping farms in New York consisted of small-scale organic vegetable gardens that produce table beet and swiss chard, intermixed with other fresh market vegetables, on an annual basis. The monoculture table beet fields consisted of broad-acre (> 0.2 km^2^) table beet fields with at least 2- to 3-year rotations with non-host crops. The *C*. *beticola* isolates from Michigan, North Dakota and Europe were obtained from personal collections (LEH and GAS). The identity of all isolates was confirmed as *C*. *beticola* using PCR primers CercoCal-beta and CercoCal-R [46].

**Table 1:** *Cercospora* isolates collected from *Beta vulgaris* in the United States of America and Europe, and characterized through genotyping-by-sequencing and microsatellites.

Population	Location	Year	Host (Variety)	N
Europe	Denmark (*n* = 5), England (*n* = 2), Germany (*n* = 5), Italy (*n* = 6), Sweden (*n* = 2), Turkey (*n* = 5)	2009–11	Sugar beet	25
Hawaii	Diamond Head community garden, Honolulu	2015	swiss chard	34
			Table beet	33
Michigan	Michigan State University Research Field	2011	Sugar beet	4
New York	Farm 1, Hector	2015	Table beet	16
	Farm 2, Phelps	2015	swiss chard	27
			Table beet (Detroit)	39
			Table beet (Touchstone Gold)	38
	Field 3, Batavia	2015	Table beet (Ruby Queen)	54
	Field 5, Mt Morris	2015	Table beet (Red Ace)	51
North Dakota	USDA Research Field	2014	Sugar beet	12
Total				333

### Microsatellite genotyping

Amplification of 12 SSR loci in the isolates from Michigan (*n* = 4), North Dakota (*n* = 12), and Europe (*n* = 25), fragment analysis, and microsatellite allele calling was conducted as described by Vaghefi et al. [37]. Fragment analysis was conducted at the Cornell University Institute of Biotechnology Genomic Diversity Facility, using a GeneScan-500 LIZ size standard (Applied Biosystems) on an ABI 3730xl DNA Analyzer. To reduce the effect of genotyping error and missing data on the results, the data were filtered in *poppr* [29,45] using a filtering threshold estimated by the function cutoff_predict (0.02083333). These data were combined with previously published data for the *C*. *beticola* isolates collected from New York (*n* = 225) and Hawaii (*n* = 67) [37].

### Genotyping-by-sequencing

DNA extraction of single-conidium-derived isolates was conducted on lyophilized mycelial tissue as described by Vaghefi et al. [36]. DNA integrity was evaluated by gel electrophoresis and quantification conducted using a Qubit fluorometer (Thermo Fisher Scientific, Waltham, MA, USA). DNA from each isolate (40–100 μl; >20 ng/μl) was submitted to the Cornell University Institute for Genomic Diversity (IGD) for DNA clean-up and GBS [23]. In brief, a reduced representation library was created by digesting genomic DNA with the restriction enzyme *Pst1*; oligonucleotide adapters were ligated onto restriction fragments; samples were pooled, enriched by PCR, and sequenced (100 bp single-end) on an Illumina Hi-Seq2500 sequencer (Illumina, San Diego, CA, USA). Due to its dependence on digestion with restriction enzymes, the GBS method developed by Elshire et al. [23] is highly sensitive to DNA sample quality as impurities may prevent complete digestion and result in lower read numbers. Therefore, a commercial kit (ZR-96 Genomic DNA Clean & Concentrator, Zymo Research, CA, USA) was used by the IGD to purify the DNA samples before library preparation.

To assess the reproducibility of GBS, three *Cercospora* isolates were genotyped six to eight times (Table 2). The same DNA sample for each of these isolates was included in each plate and sequencing run to evaluate the contribution of sequencing and SNP calling errors to polymorphism.

**Table 2:** Replicated DNA samples in genotyping-by-sequencing of *Cercospora* isolates, and genetic distance among the replicates.

Isolate^a^	Replicate	DNA plate	Sequencing run
Tb15-092	1	1	1
	2	1	1
	3	2	1
	4	2	1
	5	3	2
	6	3	2
	7	4	2
	8	4	2
Average genetic distance among replicates^b^	0.000283 (0.001855)		
Tb15-169	1	1	1
	2	1	1
	3	2	1
	4	2	1
	5	3	2
	6	4	2
Average genetic distance among replicates	0.001298 (0.004451)		
Tb15-547	1	1	1
	2	2	1
	3	3	2
	4	3	2
	5	4	2
	6	4	2
Average genetic distance among replicates	0.000101 (0.000538)		

^a^ Isolates Tb15-092 and Tb15-169 are *C*. *beticola*. Tb15-547 was later identified as *C*. cf. *flagellaris*.

^b^ Bitwise distance as estimated in *poppr* v 2.0 [45] for the SNP data set with relaxed filtering parameters. The maximum distance among replicates is given in parentheses. For the strictly filtered SNP data set, the bitwise distance among the DNA replicates was zero.

### SNP calling and strict vs. relaxed quality filtering

Genotype calling was performed by IGD using the TASSEL-GBS pipeline implemented in Tassel v. 3.0.174 [47]. In this method, only sequences that align to the reference genome (74% of the sequence tags) yield SNPs. However, SNPs are identified based on differences among the isolates and not relative to the reference genome. In brief, sequence tags (unique sequences trimmed of ambiguous nucleotides and barcodes to 64 bp) were aligned to the draft genome of *C*. *beticola* isolate Tb14-085 (collected from table beet in Batavia, New York [36]) using the Burrows-Wheeler Aligner (BWA; [48]). Only sequence tags present at least three times (pooled sample depth of three) were used to identify SNPs. The resulting variant call format (VCF) file was filtered to include only SNPs with minor allele frequency greater than 0.01 and maximum missing data of 90% by IGD. The data set obtained from the IGD was further filtered for more stringent parameters using TASSEL v. 5.2.33 [49] and Vcftools v. 0.1.14 [50] on the Linux cluster at the Cornell University BioHPC Computing Lab, Ithaca, New York, USA.

A preliminary exploratory analysis was conducted on the entire data set using principal component analysis (PCA) in TASSEL. Genotypes were converted to numeric scores and missing data was imputed to the mean score for each site. The resulting eigenvalues were visualized within a scatter plot.

As *C*. *beticola* is haploid, all heterozygous sites derived from sequencing or SNP calling errors were recoded as missing. The data set was further filtered using two approaches; i) relaxed filtering for a minimum minor allele frequency of 0.01 and a maximum of 0.25 missing data for each locus; and ii) stricter filtering for a minimum locus-by-individual (genotype) read depth of three, minimum minor allele frequency of 0.01, and a maximum of 0.10 missing data for each locus, *i*.*e*., only loci with at least 90% coverage in all isolates were retained. These two SNP data sets are referred to as the “relaxed-filtered” (S1 File) and “strictly filtered” (S2 File) data sets herein. In both data sets, genotypes with > 20% missing data were removed from the analyses. Variable sites produced by each method were converted into VCF files to enable importation to R, using the software *vcfR* [51]. Bitwise genetic distance, which calculates the fraction of different loci among samples, counting missing data as equivalent in comparison, was estimated among the replicated DNA samples for the relaxed-filtered and strictly filtered SNP data sets [29,45].

The replicated DNA samples were removed from data sets for all subsequent analyses. Removal of the genotypes with more than 20% missing data from the relaxed-filtered and strictly filtered SNP data sets resulted in a total of 307 and 310 individuals, respectively. To enable meaningful comparisons of clonal diversity indices among the data sets, the microsatellite and the strictly filtered SNP data sets were reduced to include only the 307 individuals in the relaxed-filtered SNP data set.

### Data analysis

#### Delineation of multi-locus lineages (MLLs)

Sequencing and scoring errors, somatic mutations, and missing data may inflate the number of clones by assigning individuals belonging to the same clone to separate MLGs [32]. To reduce the effect of such phenomena on identification of clones, the microsatellite and SNP data sets were contracted using distance thresholds identified in *poppr* v. 2.0 [45]. The microsatellite data was contracted using Bruvo’s distance [52] “farthest neighbour” algorithm, and a filtering threshold of 0.02083333, estimated by the *cutoff_predict* function in *poppr*. For the SNP data sets, two approaches were taken. In the first approach, the *cutoff_predict* function in *poppr* was used to estimate the distance threshold for MLL boundaries (*mlg*.*filter* threshold = 1.854599 × 10^−4^ and 0 for the relaxed-filtered and strictly filtered data sets, respectively), and all genotypes with the estimated distance threshold were collapsed into the same multi-locus lineage (MLL). In the second approach, the average bitwise distance among the replicated DNA samples was used (1.298 × 10^−3^ and 0, for the relaxed-filtered and strictly filtered data sets, respectively; Table 2) to collapse all genotypes with the calculated distance threshold into the same MLL. This resulted in three SNP data sets; 1) relaxed-filtered contracted using the more conservative distance threshold estimated by *poppr* (1.854599 × 10^−4^), herein referred to as the “relaxed-filtered data set 1”; 2) relaxed-filtered contracted using a larger distance threshold (1.298 × 10^−3^) based on the average distance among replicated DNA samples, herein referred to as the “relaxed-filtered data set 2”; and 3) strictly filtered data set contracted with the distance threshold of zero. All subsequent analyses were conducted on the contracted data sets.

For the SNP and microsatellite data sets, Nei’s measure of allelic diversity (H_e_) [53], the number of multi-locus lineages (MLLs), clonal fraction (CF), Simpson’s complement index of genotypic diversity (λ) [54] corrected for sample size, and recurrent MLLs (MLLs that occurred more than once) were obtained using *poppr*. For the microsatellite data set, allelic richness (Ra) was estimated with rarefaction in ADZE v. 1.0 [55]. Pearson’s correlation coefficient was used to assess the association between indices of clonal diversity estimated from the microsatellite and the SNP data sets. For the microsatellite data set, the probability that recurrent MLGs (MLGs that occurred more than once) could have arisen through sexual reproduction was estimated through *P*sex in GenClone 2.0 [32], and statistical significance was computed by 999 randomizations.

#### Population structure and differentiation

Jost’s measure of population differentiation (*D*) [56], pairwise Nei’s *G*_ST_ [57] and pairwise *F*_ST_ [58] were estimated using the package *mmod* [59] implemented in *adegenet* [60], and *hierfstat* [61]. The Mantel test [62] was performed using *ade4* [63] implemented in *mmod*, with 999 permutations, to quantify associations between values of *D*, *G*_ST_ and *F*_ST_ obtained from the microsatellite and SNP data sets. Discriminant analysis of principal components (DAPC) among *C*. *beticola* populations was conducted using *adegenet* [60]. The optimal number of principal components (PCs) for each data set was determined using the *xvalDapc* function.

The number of genetic clusters (*K*) and assignment of individuals to each cluster without *a priori* assumption of populations were estimated using the program, STRUCTURE [64]. Assignment of MLLs to clusters was inferred for *K* = 1–10. Each model was simulated five times with 100,000 iterations and a burn-in period of 10,000 Monte Carlo Markov Chains. The optimal number of clusters was chosen by computing Evanno’s Δ*K* [65] through STRUCTURE HARVESTER v.0.6.94 [66]. The replicated runs for the optimal *K* were combined using CLUMPAK [67] and a single graphical output was generated.

## Results

### GBS data summary

A total of 349 genotyped DNA samples had an average of 1,168,032 ± 512,427 reads that passed quality filtering. One DNA sample with less than 10,000 reads failed the quality filtering. The unfiltered data set containing all the 350 DNA samples representing 333 individuals (17 replicates of three individuals) included 27,838 SNPs, which reduced to 19,126 SNPs after initial filtering by the IGD. Further filtering of the entire data set for minor allele frequency of at least 0.01, and minimum 80% coverage of loci resulted in 7,431 SNPs.

**Fig 1:**
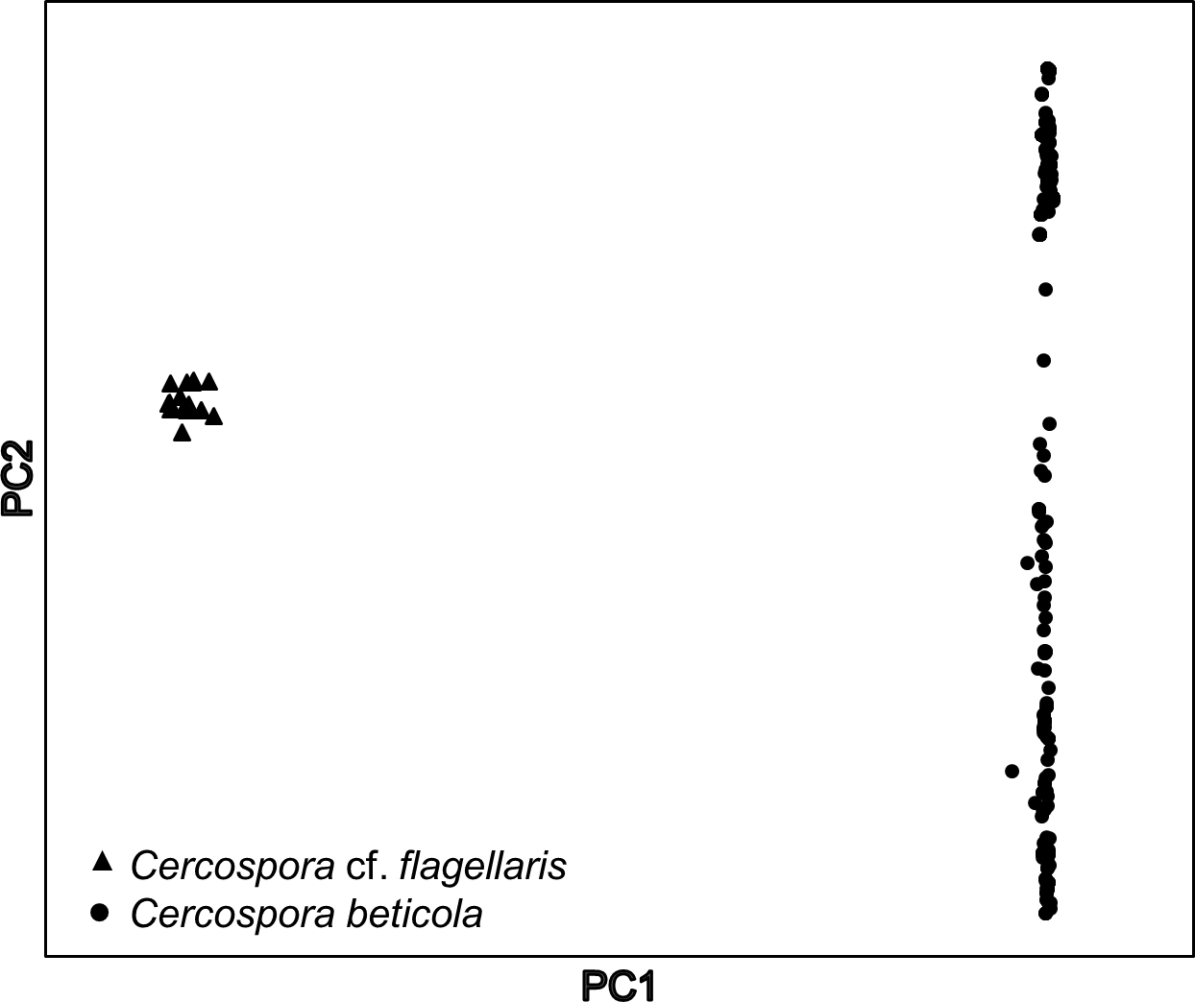
Principal component analysis of 333 *Cercospora* spp. isolates collected from *Beta vulgaris* genotyped through genotyping-by-sequencing (GBS). SNPs (n = 7,431) obtained through GBS detected two distinct clusters later identified as *C*. cf. *flagellaris* (triangles) and *C*. *beticola* (circles) using multi-locus sequence typing.

PCA using the entire data set (333 individuals and 7,431 SNPs) detected two distinct clusters of individuals (Fig 1), separating 18 DNA samples (from Fields 3 and 5) into a distinct cluster. Microsatellite loci failed to amplify in these same samples. Subsequent multi-locus phylogenetic analyses (ITS, actin, calmodulin, histone H3, and translation elongation factor 1-α) revealed these samples were a different species; *Cercospora* cf. *flagellaris*, and were not included in subsequent analyses.

### Relaxed vs. strict-filtering of SNP data set

For the relaxed-filtered data set, 2,696 SNPs were retained in 319 DNA samples (307 individuals); and 4.89% of the data set was missing. Strict filtering parameters retained 1,631 SNPs in 322 DNA samples (310 individuals), resulting in 1.35% missing data.

### Replicated samples

For the relaxed-filtered SNP data set, none of individuals had the same MLG, including the replicated samples. The bitwise distance among the replicated samples ranged from 4.1 × 10^−4^ to 1.855 × 10^−3^ (Table 2). For the strictly filtered SNP data set, the bitwise distance among the replicated DNA samples was zero. However, 37–50% of the replicated samples were identified as different MLGs, which were attributed to loci with missing data.

### Delineation of clonal lineages

Genotyping of the 307 *C*. *beticola* isolates using microsatellites resulted in detection of 130 MLGs. Contracting the microsatellite data set using the threshold estimated by the *cutoff_predict* function in *poppr* resulted in collapsing of 10 genotypes and retention of 120 MLLs (Table 3).

**Table 3:** Indices of multi-locus diversity for *Cercospora beticola* populations from Hawaii (HI), New York (NY) and Europe (EUR) after genotyping using 12 microsatellite loci [31,33] and genotyping-by-sequencing (GBS) [23]. The relaxed-filtered GBS data set included minimum minor allele frequency of 0.01 and a maximum of 25% missing data for each locus. The strictly filtered data set included a minimum locus-by-individual read depth of three, minimum minor allele frequency of 0.01, and a maximum of 10% missing data for each locus.

Population^a^	N^b^	Microsatellite loci			GBS–Relaxed-Filtered (2,696 SNPs)						GBS–Strictly Filtered (1,631 SNPs)		
								Data set 1 (mlg.filter threshold = 1.855 × 10^−4^)			Data set 2 (mlg.filter threshold = 1.298 × 10^−3^)			mlg.filter threshold = 0		
		MLL^c^	λ^d^	CF^e^	MLL^c^	λ^d^	CF^e^	MLL^c^	λ^d^	CF^e^	MLL^c^	λ^d^	CF^e^
**HI**	65	7	0.42	0.89	30	0.96	0.53	12	0.90	0.81	6	0.68	0.91
**ND**	12	8	0.89	0.33	11	0.98	0.08	10	0.97	0.17	8	0.89	0.33
**NY**–Farm 1	15	6	0.70	0.60	13	0.98	0.13	7	0.78	0.53	4	0.73	0.73
**NY**–Farm 2	98	35	0.96	0.64	82	0.99	0.16	60	0.98	0.38	34	0.96	0.65
**NY**–Field 3	47	32	0.98	0.32	41	0.99	0.13	35	0.98	0.25	29	0.97	0.38
**NY**–Field 5	43	17	0.89	0.61	36	0.99	0.16	24	0.94	0.44	15	0.88	0.65
**EUR**	23	21	0.99	0.09	22	0.99	0.04	22	0.95	0.04	20	0.98	0.13

^a^ Due to the small number of individuals from Michigan (*n* = 4), the indices of clonal diversity were not estimated for this population

^b^ N = population size

^c^ MLL = Number of multi-locus lineages after contracting the data set using the *mlg*.*filter* function in *poppr* [45]

^d^ λ = Simpson’s complement index of genotypic diversity defined as the probability that two genotypes randomly chosen from the population are different

^e^ CF = clonal fraction = (N–number of MLLs)/N.

The relaxed-filtered SNP data set (2,696 SNPs in 307 individuals) included 307 MLGs. Collapsing the data set using the more conserved threshold estimated by *cutoff_predict* resulted in 235 contracted MLLs (relaxed-filtered SNP data set 1). Using the average bitwise distance among the replicated DNA samples as the threshold resulted in 166 MLLs within the 307 individuals (relaxed-filtered SNP data set 2; Table 3).

The strictly filtered SNP data set (1,631 SNPs in 307 individuals) included 275 MLGs, *i*.*e*., 32 individuals had the same genotype at all 1,631 SNPs loci. The distance threshold estimated by the *cutoff_predict* function and maximum distance among the replicated DNA samples were estimated as zero. Therefore, the strictly filtered SNP data set was only contracted once, collapsing all the individuals with a bitwise distance of zero to the same MLL, resulting in 111 MLLs (Table 3).

### Recurrent lineages and genotype flow

**Fig 2:**
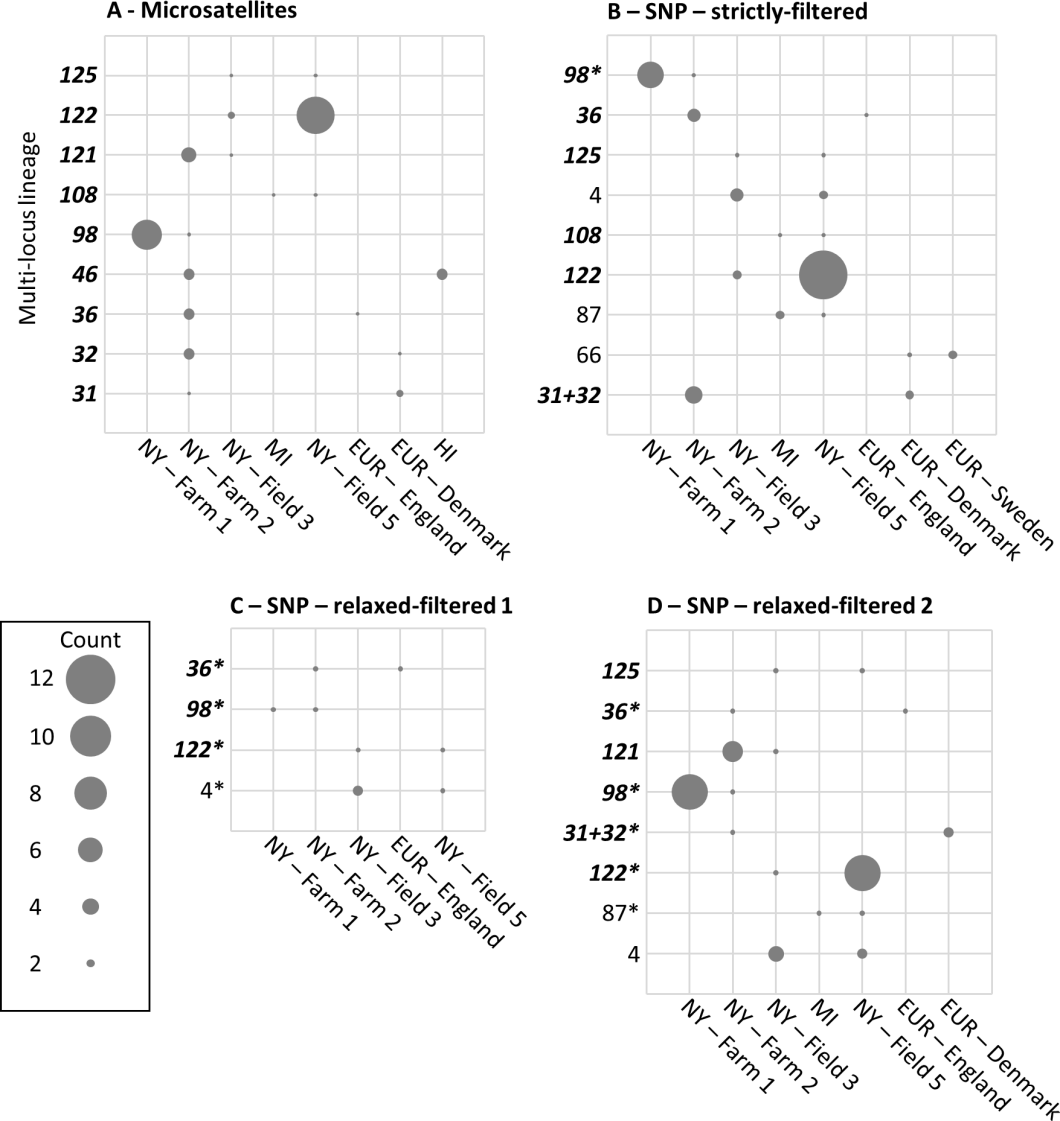
Recurrent multi-locus lineages (MLLs) shared among *Cercospora beticola* populations. **Circles represent MLLs shared among Hawaii (HI), Michigan (MI), New York (NY), and Europe (EUR), with circle sizes proportional to MLL frequencies.** The vertical axes show the MLLs detected in the microsatellite (A) and single nucleotide polymorphism (SNP) data sets generated through genotyping-by-sequencing (B [strictly filtered], C [relaxed-filtered data set 1], and D [relaxed-filtered data set 2]). MLLs detected using microsatellites are indicated in bold and italic font. When the same MLL was detected in a SNP data set, the original MLL number was replaced with the microsatellite MLL number to allow comparisons between markers. SNP MLLs that included some, but not all, of the individuals in a microsatellite MLLs are indicated with an asterisk.

Of the 120 MLLs identified using microsatellites, 43 were recurrent and nine were shared among populations (Fig 2A). MLLs 98, 121, 122 and 125 were shared among the *C*. *beticola* populations from New York. MLLs 46 and 108 were shared between different states within the USA. Three MLLs were shared between New York and Europe, all of which occurred in Farm 2 (MLLs 31, 32 and 36). The probability of the recurrent MLLs having originated from independent sexual events was ≤ 0.003 for all microsatellite MLLs.

The strictly filtered SNP data set (111 MLLs) included 46 recurrent MLLs and nine were shared among populations from different locations (Fig 2B). Four MLLs were shared among the populations within New York, and two MLLs occurred in Michigan and New York. The microsatellite MLL36 also was detected in the strictly filtered SNP data set as shared between Farm 2 (New York) and England. Microsatellite MLLs 31 and 32 were shared between Farm 2 and Denmark but not differentiated from each other in the strictly filtered SNP data set and were identified as a single clonal lineage. An additional MLL (66) was only detected in the strictly filtered SNP data set as shared between Denmark and Sweden.

Of the 235 MLLs detected in the relaxed-filtered data set 1, 46 were recurrent and four were shared among populations. Three MLLs were shared among various farms and fields within New York, while one MLL occurred in both Farm 2 and England (Fig 2C). Of the 166 MLLs in relaxed-filtered data set 2, 52 were recurrent, and eight were shared among populations; five MLLs among table beet fields and farms within New York; one MLL between New York and Michigan; and two MLLs between Farm 2 and Europe (Fig 2D).

### Indices of genetic diversity

**Fig 3:**
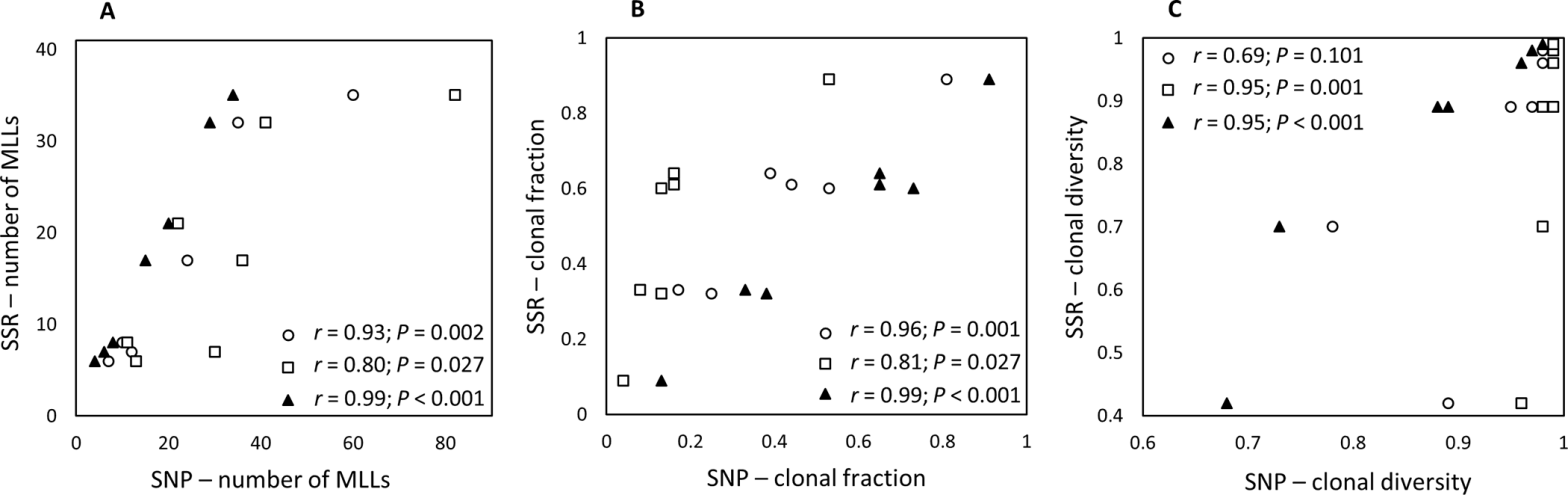
**Relationships between the (A) Number of multi-locus lineages (MLLs); (B) Clonal fraction; and (C) Simpson’s complement index of genotypic diversity for *Cercospora beticola* populations estimated using 12 microsatellites (SSR) and single nucleotide polymorphisms (SNPs) generated using genotyping-by-sequencing.** Values were estimated using the strictly filtered SNP data set (filled triangles), relaxed-filtered SNP data set 1 (open circles) and relaxed-filtered SNP data set 2 (open squares).

The indices of allelic diversity (H_e_) and richness estimated using microsatellite loci were not significantly correlated with the H_e_ obtained from the SNP data sets (r < 0.69; *P* > 0.085) (S1 Table). A significant positive correlation was detected between the number of MLLs and clonal fraction estimated from the microsatellites and SNP data sets (Fig 3A and 3B). Simpson’s complement index of genotypic diversity (λ) obtained with the microsatellite and one of the relaxed-filtered SNP data sets was not significantly correlated (Fig 3C).

### Population structure and differentiation

Pairwise indices of differentiation obtained using microsatellites showed low differentiation among the two table beet monoculture fields (Fields 3 and 5) within New York (*D* = 0.06, *G*_ST_ = 0.05, *F*_ST_ = 0.06) while the populations from the mixed-cropping farms were more differentiated from each other (*D* = 0.46, *G*_ST_ = 0.22, *F*_ST_ = 0.12) and the other two fields (*D* > 0.20, *G*_ST_ > 0.13, *F*_ST_ > 0.12; S2–S4 Tables). The *C*. *beticola* population from Europe showed low to moderate differentiation compared to populations from Farm 2 (New York) (*D* = 0.07, *G*_ST_ = 0.03, *F*_ST_ = 0.03) and North Dakota (*D* = 0.20, *G*_ST_ = 0.08, *F*_ST_ = 0.09), but higher differentiation when compared to other populations (*D* > 0.46, *G*_ST_ > 0.21, *F*_ST_ > 0.21; S2–S4 Tables).

**Fig 4:**
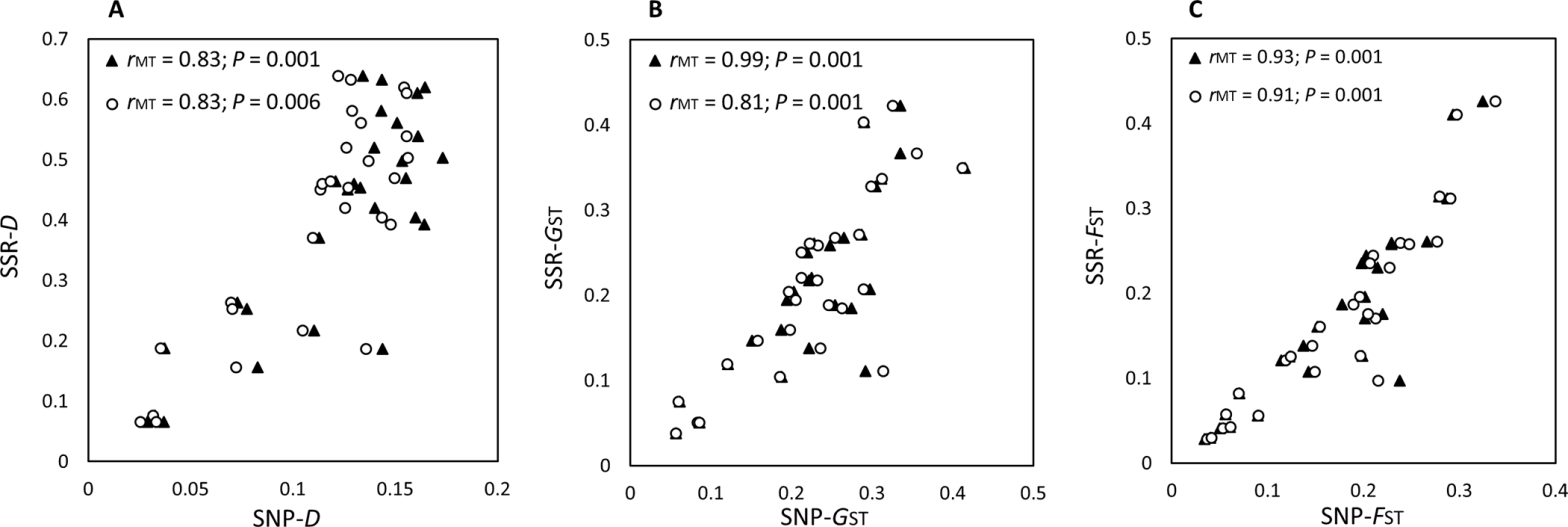
Relationships between indices of population differentiation. **(A) Jost’s *D*, (B) Pairwise Nei’s *G***_**ST,**_ **and (C) Pairwise *F***_**ST**_ **between *Cercospora beticola* populations estimated using 12 microsatellites (SSR) and single nucleotide polymorphisms (SNPs) identified using genotyping-by-sequencing.** Values were estimated using the strict SNP data set (filled triangles) and relaxed-filtered SNP data set 1 (open circles). Values estimated using the relaxed-filtered SNP data set 2 were almost identical to those obtained from data set 1.

Patterns of population differentiation obtained from SNP data sets were similar to those obtained using microsatellites (S2–S4 Tables). Mantel tests revealed strong and significant correlations between the values of pairwise *D*, *G*_ST_, and *F*_ST_ estimated by microsatellite markers and SNP data sets (Fig 4).

**Fig 5:**
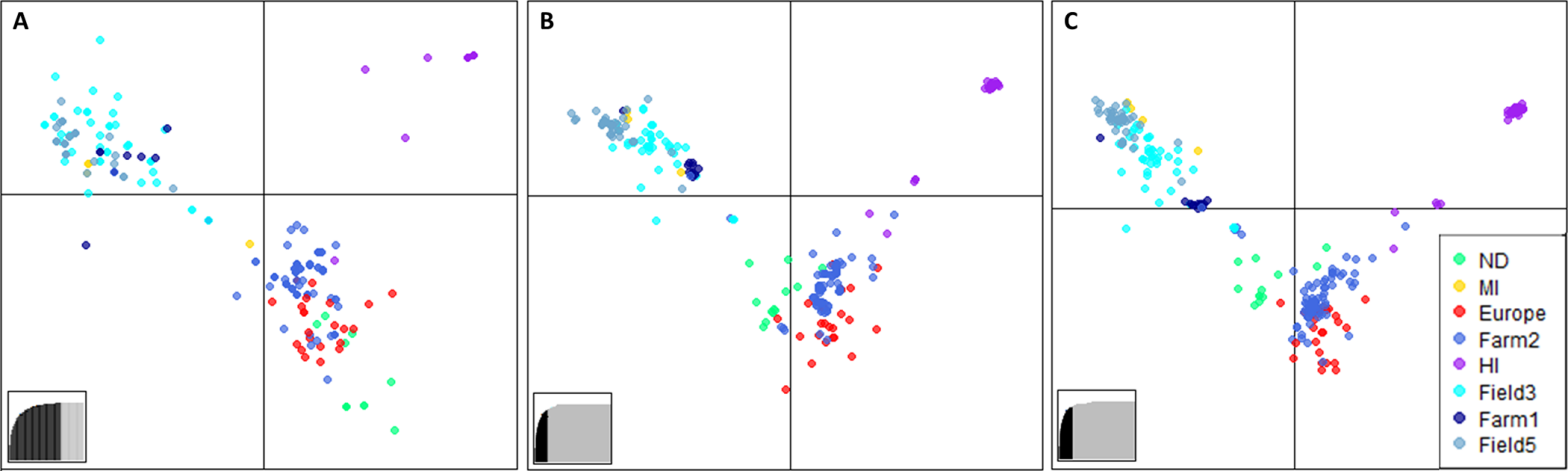
Discriminant analysis of principal components (DAPC) for *Cercospora beticola* populations from Hawaii (HI), Michigan (MI), New York (Farms 1 and 2; Fields 3 and 5), North Dakota (ND), and Europe using (A) microsatellite, (B) strictly filtered and (C) relaxed-filtered SNP data sets generated using genotyping-by-sequencing.

Patterns of population structure were not affected by marker or SNP filtering parameters (Fig 5). DAPC analysis demonstrated that *C*. *beticola* isolates from the two, monoculture table beet fields (Fields 3 and 5) and Farm 1 in New York clustered together, and individuals from Farm 2 clustered with isolates from Europe and North Dakota (Fig 5). The majority of the Hawaiian isolates formed a distinct cluster.

**Fig 6:**
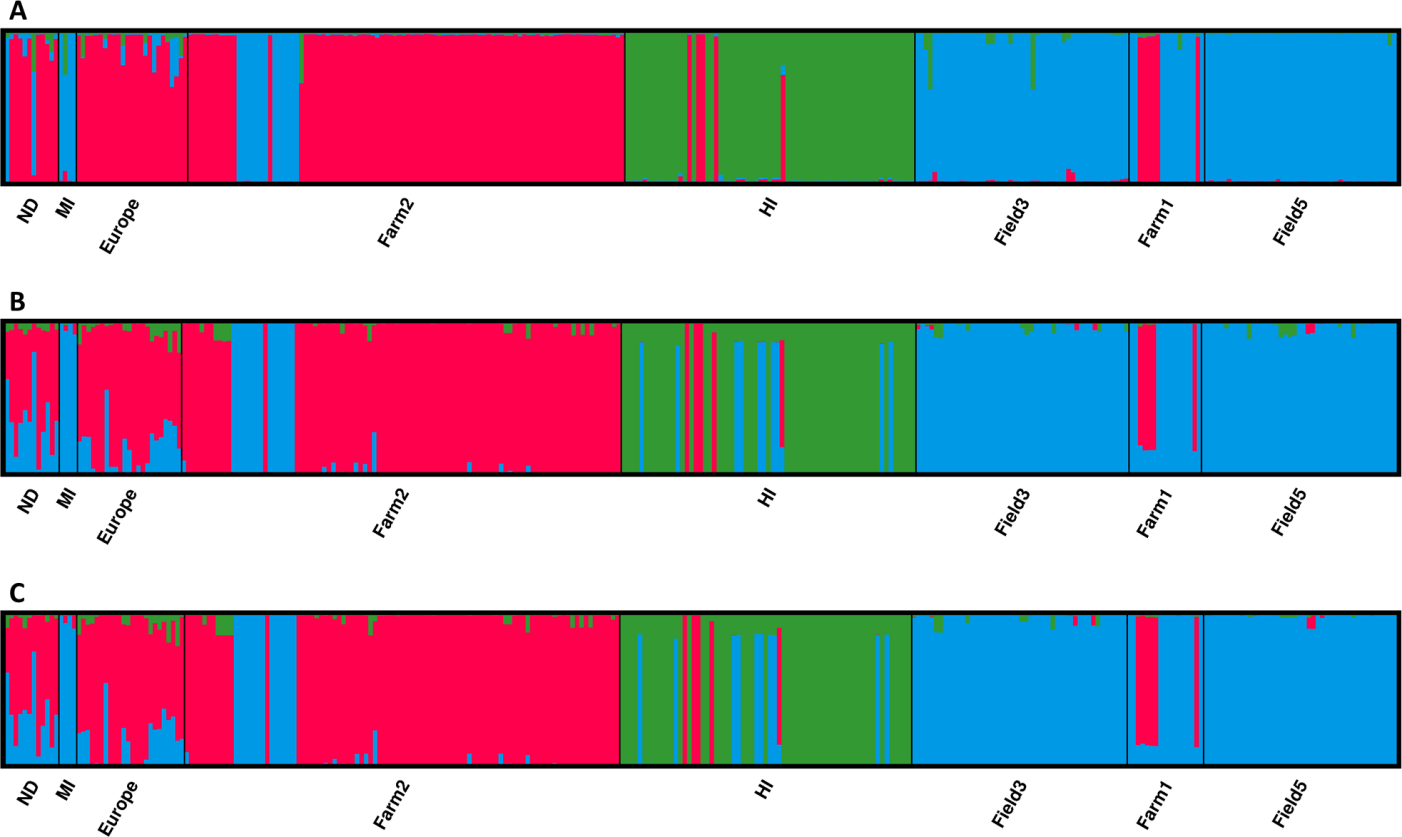
**Assignment of *Cercospora beticola* isolates from Hawaii (HI), Michigan (MI), New York (Farms 1 and 2; Fields 3 and 5), North Dakota (ND) and Europe to three clusters detected through Bayesian clustering analysis of (A) microsatellite, and single nucleotide polymorphism (SNP) data sets generated by genotyping-by-sequencing using (B) strict filtering and (C) relaxed filtering.** Each bar represents one individual and the bar height indicates estimated membership fraction of each individual in the inferred clusters.

Population structure analysis of microsatellite and SNP data sets resulted in three distinct clusters (Fig 6). The number of clusters was not affected by marker or SNP data set filtering parameters. However, assignment of some individuals to populations differed between data sets generated from the two markers (Fig 6). This could be due to hypervariability of the microsatellite loci and resultant homoplasy in MLGs that are not identical by descent depicted by state. Therefore, individuals with different SNP profiles may be assigned to the same clonal lineage when only analyzed at 12 microsatellite loci.

The three clusters detected in the microsatellite data set consisted of 60 (cluster 1; green in Fig 6A), 120 (cluster 2; blue in Fig 6A) and 127 (cluster 3; red in Fig 6A) individuals. The most distinct of the clusters was composed entirely of individuals from Hawaii (cluster 1). Five individuals from Hawaii were assigned to cluster 3 using microsatellites and SNP data sets. Analysis of the SNP data sets assigned an additional nine isolates from Hawaii to cluster 2 (Fig 6B and 6C). Cluster 2 almost exclusively included individuals from New York and Michigan, in addition to two individuals from North Dakota (Fig 6A). The SNP data sets also assigned two individuals from North Dakota to cluster 2, but their identity was not consistent between the microsatellite and the SNP data sets. Unique to the SNP data sets, was the assignment of an individual from Europe (Germany) to cluster 2. Cluster 3, was the most diverse in all data sets, and included individuals from Europe, Hawaii, New York and North Dakota.

## Discussion

Advances in high throughput SNP genotyping approaches have facilitated the identification of multitudes of SNPs in non-model organisms [19], which offer potential advantages over microsatellites for population genetics analyses due to genome-wide coverage, enhanced resolution of population diversity and structure, and stable inheritance facilitating simpler data analysis [14–18]. Our comparative analyses revealed major differences in the estimates of genetic diversity obtained from GBS and microsatellite data sets. However, both marker types and filtering parameters for the GBS data set revealed similar patterns of structure and differentiation in *C*. *beticola* populations from the USA and Europe.

The SNP data sets provided higher resolution in identification of MLGs compared to microsatellites but also greater error rates [68]. A previous study revealed that the error rates for the 12 microsatellite markers used in the current work for genotyping of *C*. *beticola* populations were zero, except for a single hypervariable locus (CbSSR3) with an error rate of 0.01 [37]. Here, we assessed GBS genotyping error through including replicated samples and estimating the mismatch rate [69]. Genetic distance among replicated DNA samples varied among isolates and was attributed to a combination of error sources including sensitivity of GBS results to DNA sample quality and quantity, sequencing error and locus drop out. Relaxed filtering of the SNP data set resulted in a bitwise distance of 4.1 × 10^−4^ to 1.855 × 10^−3^ among replicated samples. In contrast, strict filtering parameters resulted in an error rate of zero. It has been suggested that higher mismatch rates among replicates are associated with lower read depth [70]. For diploid organisms, filtering for minimum read depth from four to seven has been used to reduce error rates [25,70]. For the haploid *C*. *beticola*, filtering the SNP data set for a minimum locus-by-individual read depth of three was sufficient to reduce the mismatch rate of replicated samples to zero. Stringent filtering approaches are especially critical for population genomics analyses that rely on individual identification and delineation of clonal lineages [69–71].

Higher error rates and resolution of GBS approaches can substantially inflate the number of MLGs [12,20]. Even when the SNP data set was filtered using stringent parameters, some of the replicated samples with a mismatch rate of zero were assigned to different MLGs due to missing data. Thus, a more biologically relevant representation of clonality was obtained by collapsing MLGs to MLLs. Contraction of MLGs using a threshold that was too low resulted in inflation of the number of MLLs, and genotypic diversity in all populations was greater than 0.96 (relaxed-filtered data set 1). When the relaxed-filtered data set was contracted using an arbitrary threshold based on the distance among replicated samples, the index of genotypic diversity decreased for some populations but did not correlate with the microsatellite data or the strictly filtered SNP data set. More stringent filtering parameters reduced the number of SNPs from 2,696 to 1,361, and the indices of genotypic diversity were strongly correlated with those obtained from the microsatellite data (Fig 3). There is no “standard” way of filtering and contracting genome-wide SNP data sets, and such parameters are selected based on the type of data, objectives, and biology of the organism in question [20,71]. As filtering parameters and distance thresholds for contracting MLGs substantially impacted the number of retained loci and MLLs, we suggest that population genomics studies based on GBS use variable filtering parameters to critically assess the sensitivity of the results to the filtering approaches.

Detection of repeated MLLs of *C*. *beticola* across two continents strengthens the argument for genotype flow [36,38]. Even with relaxed-filtering of the SNP data set and a conservative distance threshold for contraction of MLGs to MLLs, which was even lower than the average error rate, one recurrent clonal lineage was shared between New York and England (Fig 2C) and multiple MLLs were shared among table beet fields in New York. The microsatellite data set (Fig 2A) and strictly filtered SNP data set (Fig 2B) detected many more MLLs that were shared between continents, and among different states in the USA.

A potential means of long distance migration of clonal lineages is contaminated seed. *Cercospora beticola* has been associated with raw sugar beet seed (‘beet balls’) [43] and population genetics studies in Europe have suggested infested seed as a potential source of inoculum [34,44]. However, other studies have not found *C*. *beticola* on sugar beet seed [72,73]; and the presence of *C*. *beticola* on table beet seed lots in New York is also unknown. Commercially available table beet seed is not polished to remove the exterior corky layer as is done in sugar beet seed to enhance germination and reduce pathogen contamination. The seed planted in the monoculture fields in New York (Fields 3 and 5) originated from Skagit Co., Washington State, USA, where occurrence of *C*. *beticola* in seed crops is rare [74]. If the high number of shared MLLs and low population differentiation of *C*. *beticola* populations from table beet monoculture fields in New York is not a result of contaminated seed, transfer by agricultural machinery [41,42] or insects [39,43] may also be involved. The shared clonal lineages between New York and Europe were collected from table beet at Farm 2, which is a mixed-cropping organic production enterprise. The table beet seed used at Farm 2 is obtained annually from various organic certified seed providers, while the seed planted at Farm 1 had been produced at that location for many years.

The predominant source of *C*. *beticola* inoculum in table beet and sugar beet fields is most likely to be alternative weedy hosts or volunteers [43,75], infested plant debris [76,77] or soilborne inoculum [78]. *Cercospora beticola* is reported to persist for 22 months to over three years on infested plant debris [76,77], and also remain virulent for 27 and 20 months in sterilized and *C*. *beticola*-infested field soil, respectively [78]. CLS epidemics then spread rapidly within a few weeks through short-range rain splash of asexual spores, which are unlikely to be involved in long-distance dispersal [39,40]. The presence of a sexual form remains unknown but multiple studies have postulated cryptic sex [79–81]. Even if *C*. *beticola* is capable of sexual reproduction, due to its heterothallic nature [79], ascospores are unlikely to be of the same clonal lineage and will most likely have recombinant genotypes [82]. Thus, ascospores are unlikely to be the source of long distance dispersal of MLLs in this study. Other plausible routes for inter-continental transmission of *C*. *beticola* is international trade of diseased alternative hosts. For example, Groenewald et al. [83] reported *C*. *beticola* from *Chrysanthemum* spp. suggesting that trade in cut flowers and ornamentals could be involved. Evidence for long distance migration of *C*. *beticola* genotypes provided here warrants further investigations on potential seedborne inoculum as a means of dispersal.

Although measures of genetic diversity (H_e_ and λ) were not associated between different marker systems, there was significant correlation among the number of clonal lineages and clonal fraction estimated from microsatellite and SNP data sets, which led to similar clonality rankings of populations across data sets. The population in Hawaii was the most clonal while the European population had the highest diversity. This is most likely an artefact of sampling strategies as the population from Hawaii was isolated from one community garden while the isolates from Europe were collected from a broad geographical area. Lack of significant correlation between expected heterozygosity obtained from microsatellites (SSR-H_e_) and SNPs (SNP-H_e_) has been reported, and may be due to the restricted genome coverage of microsatellites [13,84].

Absolute values of population differentiation (*D*, *G*_ST_ and *F*_ST_) obtained from the microsatellite and SNP data sets varied, however, there was a strong, significant correlation between the indices of differentiation, supporting results of other studies [13,84–87]. Analyses based on allele frequencies, such as estimates of genetic differentiation, are not as affected by genotyping errors as those based on individual identification [69]. Although shared recurrent MLLs were indicative of long distance dispersal of inoculum, pairwise population genetic differentiation and clustering of individuals based on geographic location indicated that the *C*. *beticola* population is not panmictic within New York, nor worldwide, providing evidence for restricted dispersal of inoculum.

Spatial patterns in *C*. *beticola* population structure were not affected by markers or filtering parameters for the SNP data set. However, minor differences were observed in the assignment of individuals to inferred clusters. Other studies have also found no or little impact of marker system on biological conclusions concerning the broad-scale structure of populations [70,88,89]. In general, due to the lower information of SNPs, accurate estimation of the number of populations has been reported to require more SNPs than microsatellites [11,24,85,89,90].

In conclusion, different marker systems, filtering approaches and distance thresholds used to collapse MLGs to MLLs, strongly affected clone identification in *C*. *beticola*. However, general patterns of variation or population structure were not affected by marker type or filtering parameters used to interrogate SNP data sets. For analyses based on allele frequencies, maximizing the number of SNPs may be more beneficial. In contrast, for analyses that require confidence in genotype of individuals, more stringent filtering for locus-by-individual read depth and clear definition of clonal boundaries based on genotyping error rate is necessary. The results also emphasize the need for development of species-specific molecular markers for rapid and reliable detection of *C*. *beticola*. The PCR markers based on the calmodulin region reliably differentiate *C*. *beticola* from *C*. *apii* [46], but failed to differentiate *C*. cf. *flagellaris* isolates collected from CLS symptoms on table beet. An earlier study also demonstrated these primers did not differentiate *C*. *chenopodii* from *C*. *beticola* [38]. The GBS-SNP data produced here may be useful for discovery of informative SNPs for diagnostic assay development [90,91].

## Supporting information

S1 Table Genetic diversity of *Cercospora beticola* populations based on 12 microsatellite (SSR) markers and single nucleotide polymorphism (SNP) data sets obtained through genotyping-by-sequencing (GBS). (DOCX)

S2 Table Genetic differentiation among *Cercospora beticola* populations based on pairwise Nei’s *G*_ST_ [57] calculated in the package *mmod* [59]. (DOCX)

S3 Table Genetic differentiation among *Cercospora beticola* populations based on pairwise *F*_ST_ [58] calculated in the package *hierfstat* [61]. (DOCX)

S4 Table Genetic differentiation among *Cercospora beticola* populations based on pairwise Jost’s *D* [56] calculated in the package *mmod* [59]. (DOCX)

S1 File Relaxed-filtered data set (VCF). (VCF)

S2 File Strictly filtered data set (VCF). (VCF)
