# Supplementary material for: Global genotype flow in Cercospora beticola populations confirmed through genotyping-by-sequencing
Source: PLoS One. 2017 Oct 24;12(10):e0186488. doi: 10.1371/journal.pone.0186488 (PMC5655429; doi:10.1371/journal.pone.0186488)
Supplement: S1 Table — (DOCX) [file pone.0186488.s001.docx]

Global Genotype Flow in *Cercospora beticola* Populations Confirmed through Genotyping-By-Sequencing

**Niloofar Vaghefi^1^, Julie R. Kikkert^2^, Melvin D. Bolton^3,5^, Linda E. Hanson^4^, Gary A. Secor^5^, Scot C. Nelson^6^, Sarah J. Pethybridge^1*^**

**1** School of Integrative Plant Science, Plant Pathology & Plant-Microbe Biology Section, Cornell University, Geneva, New York, United States of America, **2** Cornell Cooperative Extension, Canandaigua, New York, United States of America, **3** United States Department of Agriculture – Agricultural Research Service (USDA-ARS), Red River Valley Agricultural Research Center, Fargo, North Dakota, United States of America, **4** USDA-ARS, Sugar Beet and Bean Research Unit, Michigan State University, Michigan, United States of America, **5** Department of Plant Pathology, North Dakota State University, Fargo, North Dakota, United States of America, **6** College of Tropical Agriculture and Human Resources, Department of Tropical Plant and Soil Sciences, University of Hawaii at Manoa, Honolulu, Hawaii, United States of America

*[sjp277@cornell.ed.au](mailto:sjp277@cornell.ed.au) (SJP)

**Table S1. Genetic diversity of *Cercospora* *beticola* populations based on 12 microsatellite (SSR) markers and Single nucleotide Polymorphism (SNP) data sets obtained through genotyping-by-sequencing (GBS).** Nei’s measure of allelic diversity (H_e_) [52] was estimated for both markers, and allelic richness (R_a_) was estimated with rarefaction in ADZE [54] for the microsatellite data set.

| **Diversity indices**  **Population** | **SSR-H_e_** | **SSR-R_a_** | **SNP-H_e_** | |
| --- | --- | --- | --- | --- |
|  |  |  | **Strictly-Filtered**  **(1,631 SNPs)** | **Relaxed-Filtered**  **(2,696 SNPs)** |
| **North Dakota** | 0.564 | 3.396 | 0.00070 | 0.00056 |
| **Europe** | 0.541 | 4.167 | 0.00134 | 0.00234 |
| **Hawaii** | 0.245 | 2.833 | 0.00011 | 0.00087 |
| **New York – Farm 1** | 0.448 | 2.583 | 0.00000 | 0.00000 |
| **New York – Farm 2** | 0.461 | 4.083 | 0.00063 | 0.00221 |
| **New York – Field 3** | 0.399 | 4.583 | 0.00041 | 0.00085 |
| **New York – Field 5** | 0.365 | 3.000 | 0.00030 | 0.00018 |
