# Supplementary material for: Global genotype flow in Cercospora beticola populations confirmed through genotyping-by-sequencing
Source: PLoS One. 2017 Oct 24;12(10):e0186488. doi: 10.1371/journal.pone.0186488 (PMC5655429; doi:10.1371/journal.pone.0186488)
Supplement: S2 Table — (DOCX) [file pone.0186488.s002.docx]

Global Genotype Flow in *Cercospora beticola* Populations Confirmed through Genotyping-By-Sequencing

**Niloofar Vaghefi^1^, Julie R. Kikkert^2^, Melvin D. Bolton^3,5^, Linda E. Hanson^4^, Gary A. Secor^5^, Scot C. Nelson^6^, Sarah J. Pethybridge^1*^**

**1** School of Integrative Plant Science, Plant Pathology & Plant-Microbe Biology Section, Cornell University, Geneva, New York, United States of America, **2** Cornell Cooperative Extension, Canandaigua, New York, United States of America, **3** United States Department of Agriculture – Agricultural Research Service (USDA-ARS), Red River Valley Agricultural Research Center, Fargo, North Dakota, United States of America, **4** USDA-ARS, Sugar Beet and Bean Research Unit, Michigan State University, Michigan, United States of America, **5** Department of Plant Pathology, North Dakota State University, Fargo, North Dakota, United States of America, **6** College of Tropical Agriculture and Human Resources, Department of Tropical Plant and Soil Sciences, University of Hawaii at Manoa, Honolulu, Hawaii, United States of America

*[sjp277@cornell.ed.au](mailto:sjp277@cornell.ed.au) (SJP)

**Table S2. Genetic differentiation among *Cercospora* *beticola* populations based on pairwise Nei’s *G*_ST_ [57] calculated in the package *mmod* [59].** The first number indicates *G*_ST_ calculated from genotyping 12 microsatellite loci; the second number was obtained from the strictly filtered GBS-SNP data set; and the third and fourth numbers were obtained from the relaxed-filtered GBS-SNP data set 1 and 2, respectively.

|  | **North Dakota** | **Europe** | **New York – Farm 2** | **Hawaii** | **New York – Field 3** | **New York – Farm 1** |
| --- | --- | --- | --- | --- | --- | --- |
| **Europe** | 0.0809  0.0621  0.0601  0.0601 |  |  |  |  |  |
| **New York – Farm 2** | 0.1163  0.1207  0.1199  0.1199 | 0.0335  0.0563  0.0567  0.0567 |  |  |  |  |
| **Hawaii** | 0.3282  0.3042  0.2986  0.2986 | 0.2700  0.2866  0.2839  0.2839 | 0.3322  0.3115  0.3121  0.3121 |  |  |  |
| **New York – Field 3** | 0.2058  0.2033  0.1968  0.1968 | 0.2541  0.2197  0.2123  0.2123 | 0.2566  0.2284  0.2227  0.2227 | 0.4085  0.2901  0.2892  0.2892 |  |  |
| **New York – Farm 1** | 0.1670  0.1972  0.1984  0.1984 | 0.2078  0.2057  0.2050  0.2050 | 0.2233  0.2312  0.2320  0.2320 | 0.3803  0.3411  0.3556  0.3556 | 0.1403  0.1470  0.1577  0.1577 |  |
| **New York – Field 5** | 0.2208  0.2241  0.2124  0.2124 | 0.2624  0.2469  0.2328  0.2328 | 0.2612  0.2647  0.2541  0.2541 | 0.4223  0.3342  0.3251  0.3251 | 0.0504  0.0852  0.0853  0.0853 | 0.1332  0.2218  0.2359  0.2359 |
