# Supplementary material for: Global genotype flow in Cercospora beticola populations confirmed through genotyping-by-sequencing
Source: PLoS One. 2017 Oct 24;12(10):e0186488. doi: 10.1371/journal.pone.0186488 (PMC5655429; doi:10.1371/journal.pone.0186488)
Supplement: S3 Table — (DOCX) [file pone.0186488.s003.docx]

Global Genotype Flow in *Cercospora beticola* Populations Confirmed through Genotyping-By-Sequencing

**Niloofar Vaghefi^1^, Julie R. Kikkert^2^, Melvin D. Bolton^3,5^, Linda E. Hanson^4^, Gary A. Secor^5^, Scot C. Nelson^6^, Sarah J. Pethybridge^1*^**

**1** School of Integrative Plant Science, Plant Pathology & Plant-Microbe Biology Section, Cornell University, Geneva, New York, United States of America, **2** Cornell Cooperative Extension, Canandaigua, New York, United States of America, **3** United States Department of Agriculture – Agricultural Research Service (USDA-ARS), Red River Valley Agricultural Research Center, Fargo, North Dakota, United States of America, **4** USDA-ARS, Sugar Beet and Bean Research Unit, Michigan State University, Michigan, United States of America, **5** Department of Plant Pathology, North Dakota State University, Fargo, North Dakota, United States of America, **6** College of Tropical Agriculture and Human Resources, Department of Tropical Plant and Soil Sciences, University of Hawaii at Manoa, Honolulu, Hawaii, United States of America

*[sjp277@cornell.ed.au](mailto:sjp277@cornell.ed.au) (SJP)

**Table S3. Genetic differentiation among *Cercospora* *beticola* populations based on pairwise *F*_ST_ [58] calculated in the package *hierfstat* [61].** The first number indicates *F*_ST_ calculated from genotyping 12 microsatellite loci; the second number was obtained from the strictly filtered GBS-SNP data set; and the third and fourth numbers were obtained from the relaxed-filtered GBS-SNP data set 1 and 2, respectively.

|  | **North Dakota** | **Europe** | **New York – Farm 2** | **Hawaii** | **New York - Field3** | **New York - Farm1** |
| --- | --- | --- | --- | --- | --- | --- |
| **Europe** | 0.0896  0.0715  0.0703  0.0703 |  |  |  |  |  |
| **New York - Farm2** | 0.0577  0.0567  0.0562  0.0562 | 0.0261  0.0406  0.0398  0.0398 |  |  |  |  |
| **Hawaii** | 0.2591  0.2386  0.2293  0.2293 | 0.2539  0.2759  0.2663  0.2663 | 0.3112  0.2907  0.2875  0.2875 |  |  |  |
| **New York - Field3** | 0.1636  0.1559  0.1518  0.1518 | 0.2445  0.2097  0.2026  0.2026 | 0.2333  0.2062  0.1978  0.1978 | 0.4151  0.2978  0.2933  0.2933 |  |  |
| **New York - Farm1** | 0.1847  0.2160  0.2197  0.2197 | 0.2098  0.2059  0.2016  0.2016 | 0.1219  0.1189  0.1142  0.1142 | 0.3165  0.2774  0.2786  0.2786 | 0.1173  0.1176  0.1225  0.1225 |  |
| **New York - Field5** | 0.1871  0.1885  0.1775  0.1775 | 0.2582  0.2459  0.2292  0.2292 | 0.2285  0.2265  0.2146  0.2146 | 0.4259  0.3365  0.3241  0.3241 | 0.0554  0.0901  0.0897  0.0897 | 0.1187  0.1924  0.1988  0.1988 |
