# Supplementary material for: Global genotype flow in Cercospora beticola populations confirmed through genotyping-by-sequencing
Source: PLoS One. 2017 Oct 24;12(10):e0186488. doi: 10.1371/journal.pone.0186488 (PMC5655429; doi:10.1371/journal.pone.0186488)
Supplement: S4 Table — (DOCX) [file pone.0186488.s004.docx]

Global Genotype Flow in *Cercospora beticola* Populations Confirmed through Genotyping-By-Sequencing

**Niloofar Vaghefi^1^, Julie R. Kikkert^2^, Melvin D. Bolton^3,5^, Linda E. Hanson^4^, Gary A. Secor^5^, Scot C. Nelson^6^, Sarah J. Pethybridge^1*^**

**1** School of Integrative Plant Science, Plant Pathology & Plant-Microbe Biology Section, Cornell University, Geneva, New York, United States of America, **2** Cornell Cooperative Extension, Canandaigua, New York, United States of America, **3** United States Department of Agriculture – Agricultural Research Service (USDA-ARS), Red River Valley Agricultural Research Center, Fargo, North Dakota, United States of America, **4** USDA-ARS, Sugar Beet and Bean Research Unit, Michigan State University, Michigan, United States of America, **5** Department of Plant Pathology, North Dakota State University, Fargo, North Dakota, United States of America, **6** College of Tropical Agriculture and Human Resources, Department of Tropical Plant and Soil Sciences, University of Hawaii at Manoa, Honolulu, Hawaii, United States of America

*[sjp277@cornell.ed.au](mailto:sjp277@cornell.ed.au) (SJP)

**Table S4. Genetic differentiation among *Cercospora* *beticola* populations based on pairwise Jost’s *D* [56] calculated in the package *mmod* [59].** The first number indicates *D* calculated from genotyping 12 microsatellite loci; the second number was obtained from the strictly filtered GBS-SNP data set; and the third and fourth numbers were obtained from the relaxed-filtered GBS-SNP data set 1 and 2, respectively.

|  | **North Dakota** | **Europe** | **New York – Farm 2** | **Hawaii** | **New York – Field 3** | **New York – Farm 1** |
| --- | --- | --- | --- | --- | --- | --- |
| **Europe** | 0.2011  0.0376  0.0355  0.0355 |  |  |  |  |  |
| **New York – Farm 2** | 0.2605  0.0728  0.0697  0.0697 | 0.0674  0.0323  0.0317  0.0317 |  |  |  |  |
| **Hawaii** | 0.6196  0.1644  0.1541  0.1541 | 0.4626  0.1557  0.1497  0.1497 | 0.5377  0.1611  0.1555  0.1555 |  |  |  |
| **New York – Field 3** | 0.4508  0.1269  0.1134  0.1134 | 0.5828  0.1433  0.1290  0.1290 | 0.5132  0.1400  0.1261  0.1261 | 0.6461  0.1339  0.1221  0.1221 |  |  |
| **New York – Farm 1** | 0.3778  0.1181  0.1098  0.1098 | 0.4856  0.1275  0.1184  0.1184 | 0.4621  0.1374  0.1271  0.1271 | 0.6228  0.1613  0.1555  0.1555 | 0.2298  0.0734  0.0702  0.0702 |  |
| **New York – Field 5** | 0.4600  0.1290  0.1142  0.1142 | 0.5684  0.1503  0.1333  0.1333 | 0.4905  0.1528  0.13693  0.13693 | 0.6321  0.1427  0.1283  0.1283 | 0.0644  0.0367  0.0333  0.0333 | 0.2016  0.1078  0.1046  0.1046 |
